# The Association of Recurrent and Multiple Types of Abuse with Adverse Mental Health, Substance Use, and Sexual Health Outcomes among Out-of-School Adolescent Girls and Young Women in Cape Town, South Africa

**DOI:** 10.3390/ijerph182111403

**Published:** 2021-10-29

**Authors:** Bronwyn Myers, Felicia A. Browne, Tara Carney, Tracy Kline, Courtney Peasant Bonner, Wendee M. Wechsberg

**Affiliations:** 1 Curtin enAble Institute, Faculty of Health Sciences, Curtin University, Perth, WA 6102, Australia; 2 Alcohol, Tobacco and Other Drug Abuse Research Unit, South African Medical Research Council, P.O. Box 19070, Tygerberg 7505, South Africa; tcarney@mrc.ac.za; 3 Division of Addiction Psychiatry, Department of Psychiatry and Mental Health, University of Cape Town, Cape Town 7701, South Africa; 4 RTI International, 3040 Cornwallis Road, Research Triangle Park, NC 27709, USA; fbrowne@rti.org (F.A.B.); tkline@rti.org (T.K.); cpbonner@rti.org (C.P.B.); wmw@rti.org (W.M.W.); 5 Maternal and Child Health Department, Gillings School of Global Public Health, University of North Carolina, Chapel Hill, NC 27514, USA; 6 Department of Psychology, North Carolina State University, Raleigh, NC 27529, USA; 7 Psychiatry and Behavioral Sciences, Duke University School of Medicine, Durham, NC 27703, USA

**Keywords:** young women, mental health, physical and sexual abuse, gender-based violence, substance use, trauma-informed interventions, low- and middle-income countries

## Abstract

Although physical and sexual abuse exposure is a well-established risk for poor health, the dimensions of abuse associated with health among socially vulnerable adolescent girls and young women (AGYW) remain underexamined. This article describes associations between combinations of abuse type and timing with mental health, substance use, and sexual risk outcomes among a sample of 499 AGYW (aged 16 to 19) who had left school early and were recruited for a cluster randomized trial in Cape Town, South Africa. Approximately one-third (33.5%; 95% CI: 28.7, 38.6) of participants reported lifetime abuse. Exposure to more than one type of abuse was associated with increased risk of depression (β = 3.92; 95% CI: 2.25, 5.59) and anxiety (β = 3.70; 95% CI: 2.11, 5.28), and greater odds of polydrug use (OR = 2.10; 95% CI: 1.02, 4.34) and substance-impaired sex (OR = 2.17; 95% CI: 1.31, 3.86). Exposure to multiple types of abuse during childhood/early adolescence and again in late adolescence was associated with increased risk of depression (β = 4.65; 95% CI: 3.15, 6.14), anxiety (β = 4.35; 95% CI: 2.70, 6.02), and polydrug use (OR = 2.37; 95% CI: 1.03, 5.73). Findings underscore the need for trauma-informed interventions that reduce mental health, substance use, and sexual risks among AGYW who have experienced multiple forms of abuse and recurrent abuse.

## 1. Introduction

Like many low- and middle-income countries (LMICs) [1,2], mental health concerns are highly prevalent among South African adolescents and young adults between the ages of 15 and 24, with an estimated 30% to 40% of this age cohort being at elevated risk of depression, anxiety, or problematic substance use [3,4,5,6]. Compared with their male age counterparts, adolescent girls and young women (AGYW) appear particularly vulnerable for mental health concerns [3,4,7]. This heightened vulnerability is a product of AGYW’s exposure to multiple social determinants of mental ill-health, including adverse living conditions characterized by material deprivation, low education outcomes, lack of economic opportunities, gendered experiences (such as leaving school before completion because of family caregiving responsibilities and unintended pregnancy), and exposure to violence [3,7,8] arising from widespread economic and gender inequality in South Africa [9]. Problematic substance use often co-occurs with, and heightens risk for, these mental health concerns [10]. In a context where AGYW face limited access to age- and gender-appropriate mental health and substance use treatment services [11,12,13], AGYW may use substances to manage these environmental stressors and cope with symptoms of depression, anxiety, and trauma [14,15,16,17].

If left unaddressed, these mental health and substance use concerns may have lifelong health and socioeconomic sequelae for AGYW. Untreated mental and substance use concerns during adolescence increase risk for mental disorders in adulthood [18,19], as well as noncommunicable and communicable diseases [20]. Risk of HIV acquisition is of particular concern for South Africa, with a quarter of the country’s estimated 270,000 incident HIV infections occurring among AGYW [21]. The impact of mental health and substance use concerns on sexual risk for HIV and incident infections is well documented for AGYW in this setting [22,23,24,25,26,27]. Evidence suggests that these problems may decrease AGYWs’ sexual agency, compromise adaptive coping, and contribute to suboptimal decision-making during sex, making it more difficult to resist pressure to have sex and to negotiate condom use, should they have the skills to do so [23,25,26,27,28].

These prior findings underscore the potential value of incorporating early interventions to prevent or delay the onset of mental health and substance use disorders into HIV prevention programing for AGYW [29,30]. At present, these low-threshold interventions are absent from the mental health and substance use service landscape [13,31]. Given South Africa’s constrained resources for mental health, any efforts to address this service gap should promote the efficient use of resources [13,32,33]. Transdiagnostic interventions that target shared risk factors for a range of mental and behavioral health problems are likely to offer a more efficient solution to this service gap than early interventions targeting a single problem [29,30].

Adverse childhood experiences—including racial discrimination, socioeconomic adversity, and physical and sexual abuse—are well-established transdiagnostic risks for mental health and substance use concerns in adolescence and early adulthood [34,35], with exposure to multiple forms of abuse further elevating risk [36]. Additionally, prior studies have reported direct effects of physical and sexual abuse on sexual risk for HIV, including having multiple partners, condomless sex, and substance use before or during sex [37,38,39]. Developmental timing of abuse may also impact mental health symptom severity, with some studies documenting sensitive developmental periods when exposure to abuse increases risk of poor mental health [40,41], and other studies noting the cumulative effect of abuse across multiple developmental periods on mental health outcomes [42,43]. Despite this evidence, knowledge gaps remain.

The relationship between multiple types of abuse exposure and recurrence of abuse on mental health outcomes remains underexplored, with available evidence being inconclusive and contradictory [41,44,45]. Furthermore, none of these earlier studies examined mental health vulnerabilities among out-of-school and sexually active AGYW, a key population at risk for HIV [23,26]. Understanding the dimensions of abuse that heighten vulnerability for mental health and substance use concerns is important for identifying AGYW who may benefit most from these early interventions.

This article contributes to the emerging literature on how combinations of abuse type and timing contribute to mental health, substance use, and sexual risk among out-of-school AGYW. More specifically, it aims to explore associations between abuse type and developmental timing of abuse with mental health, substance use, and sexual risks for HIV in a sample of socially vulnerable AGYW in Cape Town, South Africa.

## 2. Materials and Methods

This article analyzes baseline data from the Young Women’s Health CoOp (YWHC) study, a cluster randomized trial comparing the efficacy of the YWHC intervention with standard HIV counselling and testing (HCT) for reducing substance use and HIV risk behaviors among AGYW with increased risk for HIV in Cape Town, South Africa (Trial registration number: NCT02974998). The trial methods are described fully elsewhere [46].

### 2.1. Participants and Procedures

Between November 2016 and November 2018, 500 AGYW were recruited from 24 disadvantaged and geographically distinct communities (clusters) in Cape Town. Peers (women aged 18 to 24 who lived in the selected communities) worked together with the study outreach staff to market the study in parts of these communities with high population density, and where young women congregated, such as hair salons. Marketing involved posting study fliers, distributing recruitment cards, and talking to young women and other community members about the study. Potential participants were individually and privately screened for eligibility by outreach staff [46].

Young women were eligible for study inclusion if they were aged 16 to 19, lived in a study community, reported consuming at least two alcoholic drinks or other drug use on a weekly basis over the past 90 days, reported having discontinued school for at least 6 months, were not currently in school and had not completed school, and reported condomless sex with a male partner in the past 90 days. Young women who were eligible and interested in participating were provided with transportation to the study’s research site for study enrollment. At this appointment, study staff confirmed the participant’s eligibility before obtaining their written informed consent for study participation. Women who were aged 16 or 17 provided written informed assent to participate, with consent for their participation being obtained in loco parentis by a trusted woman from their community who was at least 25 years of age [46]. After obtaining assent/consent, field staff administered a baseline computer-assisted personal interview in English, Afrikaans, or isiXhosa, the three official languages of the province. This questionnaire collected information on sociodemographic characteristics, alcohol and other drug use, mental health, and HIV risk behavior. Participants completed sensitive questions about physical and sexual abuse via audio computer-assisted self-interviewing (ACASI) for which field staff were not present.

After this interview, field staff collected urine samples to test for rapid illicit drug and pregnancy screening. A breathalyzer was used to assess recent alcohol use. We used a 5-panel drug screening test to detect cocaine, methamphetamines, amphetamine, opioid, and cannabis metabolites, and a single panel test to assess for methaqualone (Mandrax) use. After completing this appointment, participants were provided with transport to their neighborhood, and were given a gift card to thank them for their time [46].

### 2.2. Measures

#### 2.2.1. Physical and Sexual Abuse

The following questions assessed lifetime experiences of physical and/or sexual abuse: “Has anyone ever physically abused you (for example, hurt you to the point that you had bruises, cuts, or broken bones)?” and “Has anyone ever pressured or forced you to have sex against your will?” Responses to these two questions were used to create an exposure to lifetime abuse variable, with the following responses: no abuse; physical abuse without sexual abuse; sexual abuse without physical abuse; and both physical and sexual abuse.

To better understand temporal associations between exposure to physical and sexual abuse and symptoms of mental ill-health, we examined the developmental timing of abuse. Developmental timing of abuse was assessed through items examining the age at which physical and/or sexual abuse first occurred and abuse in the 6 months prior to study enrollment. Given the small number of participants reporting physical and/or sexual abuse before the age of 16 (*n* = 51), we created a single variable to capture any abuse during childhood or early adolescence. Abuse during childhood or early adolescence was considered present when participants reported any abuse exposure before the age of 16. Abuse during late adolescence was considered present when participants reported abuse in the 6 months prior to study enrollment. Recurrent exposure was defined as exposure to abuse during both childhood/early adolescence and late adolescence.

#### 2.2.2. Mental Health, Substance Use, and Sexual Risk Variables

We used the 7-item Texas Christian University (TCU) depression scale to assess depression symptom frequency in the 90 days prior to study enrollment [47]. Originally developed for use in the Drug Abuse Treatment Outcome Study (DATOS), this scale has been adapted for use with South African populations [48]. Item responses range from never (0) to always (4), and are summed to yield a composite score (ranging from 0 to 28), with higher scores indicating greater risk of depression [47]. We used the 7-item Texas Christian University (TCU) anxiety scale to assess anxiety symptom severity in the 90 days prior to study enrollment [47]. This scale has been adapted for use with South African populations [48]. Item responses range from never (0) to always (4), and are summed to yield a composite score (ranging from 0 to 28), with higher scores indicating greater risk [47]. The depression and anxiety scales were modified by using language more relevant to the South African linguistic lexicon (e.g., changing “elevator” to “lift”) and removing language that confused participants during pre-testing of the questionnaires (e.g., “brood”). The process of pre-testing is described elsewhere, but briefly involved cognitive testing of the instrumentation with AGYW who met study inclusion criteria, and iteratively adapting the wording of problematic questionnaire items to enhance item comprehension in the South African cultural context [48]. In the YWHC data, the internal consistency estimates (Cronbach’s alpha) were 0.70 for depression and 0.80 for anxiety.

The results from drug testing were used to assess for recent polydrug use—a pattern of drug use associated with elevated risk of mental and physical health problems, including risk for HIV [26,49]. Polydrug use was coded as present (testing positive for at least two substances) or absent. Substance-impaired sex, a risk for condomless sex [50], was assessed through a single item, “The last time you had sex did you use drugs or alcohol just before or during sex?”

### 2.3. Data Analysis

All analyses were conducted using Stata 16, with statistical significance set at *p* < 0.05. We used Stata’s survey analysis platform to account for clustering by community. Using a complete case analysis, a total of 499 of the 500 participant observations were included. We estimated the prevalence (95% confidence intervals [CIs]) of each combination of abuse type for each developmental period and across the lifespan. Next, we conducted a series of multivariate linear and logistic regression models to estimate associations between each mental health, substance use, and sexual risk outcome and type, combination, and timing of abuse exposure. All models adjusted for participant age and race. We first estimated the effect of any abuse exposure on each outcome (Model 1). Following this, we estimated: the effect of type of abuse (Model 2); a combination of type of abuse (Model 3); and the timing of abuse—childhood/early adolescence, late adolescence, or both developmental periods (Model 4). Finally, we examined the effect of both combinations of type of abuse and timing of abuse (Model 5). Each model was estimated for risk of depression, anxiety, polydrug use, and substance-impaired sex. The findings are presented as odds ratios (OR) or β coefficients with 95% CI.

## 3. Results

Overall, 33.5% (95% CI: 28.7, 38.6) of the sample reported experiencing any lifetime physical and/or sexual abuse, 21.4% (95% CI: 17.9, 25.5) of the overall sample reported abuse during childhood or early adolescence, and 23.8% (95% CI: 19.6, 30.7) reported abuse during late adolescence. Irrespective of developmental period, physical abuse was more frequently reported than sexual abuse (Table 1). Overall, 8.0% (95% CI: 6.1, 10.4) of participants reported lifetime exposure to more than one form of abuse.

### 3.1. Associations between Type and Combinations of Abuse and Mental Health, Substance Use, and Sexual Risk Outcomes

In regression analyses examining associations between any abuse exposure and mental health, substance use, and sexual risks (Model 1, Table 2), any abuse exposure was associated with greater risk of depression (β = 1.52, 95% CI: 0.44, 2.60) and anxiety (β = 1.89, 95% CI: 0.67, 3.11), in addition to greater odds of polydrug use (OR = 1.57, 95% CI: 1.02, 2.52) and substance-impaired sex (OR = 1.64, 95% CI: 1.13, 2.41). In models of associations between type of abuse exposure and mental health, substance use, and sexual risk (Model 2, Table 2), both physical abuse and sexual abuse were associated with greater risk of depression (β = 1.52, 95% CI: 0.31, 2.80 and β = 1.40, 95% CI: 0.17, 2.63, respectively) and anxiety (β = 1.65, 95% CI: 0.18, 3.12 and β = 1.59, 95% CI: 0.44, 2.74, respectively). Only physical abuse was associated with greater odds of polydrug use (OR = 1.68, 95% CI: 1.04, 2.74) and engaging in substance-impaired sex (OR = 1.59, 95% CI: 1.00, 2.53).

In models examining combinations of abuse (Model 3), exposure to both physical and sexual abuse was associated with heightened risk of depression (β = 3.92, 95% CI: 2.25, 5.59) and anxiety (β = 3.70, 95% CI: 2.11, 5.28), and with greater odds of polydrug use (OR = 2.10, 95% CI: 1.02, 4.34) and substance-impaired sex (OR = 2.17, 95% CI: 1.31, 3.86). Exposure to one form of abuse, whether physical or sexual, was not significantly associated with any of the four outcomes (Table 2).

### 3.2. Associations between Developmental Period of Abuse and Mental Health, Substance Use, and Sexual Risk Outcomes

Among participants who reported exposure to any type of abuse, 28.6% (95% CI: 23.1, 34.8) reported abuse during childhood or early adolescence only, 35.7% (95% CI: 27.6, 44.8) reported abuse during late adolescence only, and 35.1% (95% CI: 27.2, 43.9) reported abuse in both developmental periods. Table 3 presents associations between developmental period of abuse and mental health, substance use, and sexual risk. The findings suggest that exposure to abuse during both developmental periods is associated with greater risk of depression (β = 3.09, 95% CI: 1.95, 4.23) and anxiety (β = 3.23, 95% CI: 1.82, 4.64), and greater odds of being substance-impaired during sex (OR = 2.47, 95% CI: 1.51, 4.07). Exposure to abuse during late adolescence (but not during childhood or early adolescence) doubled the odds of polydrug use (OR = 2.13, 95% CI: 1.31, 3.76).

### 3.3. Associations between Type and Developmental Period of Abuse and Mental Health, Substance Use, and Sexual Risk Outcomes

We estimated a model that includes type and developmental period of abuse (Table 4). The findings suggest that being exposed to *both* physical and sexual abuse in childhood or early adolescence and again during late adolescence increases risk of depression (β = 4.65, 95% CI: 3.15, 6.14) and anxiety (β = 4.35, 95% CI: 2.70, 6.02). When type and timing of abuse exposure were considered together, exposure to physical and/or sexual abuse during late adolescence (in the absence of childhood abuse) did not heighten mental health or sexual risk. Exposure to physical abuse during this developmental period was associated with greater odds of polydrug use (OR = 2.70, 95% CI: 1.30, 5.61). Exposure to abuse during childhood/early adolescence in the absence of abuse during late adolescence was associated with greater risk of depression (β = 4.52, 95% CI: 0.26, 8.78) and anxiety (β = 4.45, 95% CI: 2.66, 6.23).

## 4. Discussion

Although physical and sexual abuse are known risks for mental health and substance use concerns among young people [34,35,36], studies have rarely explored how type, timing, and multiplicity of exposures affect mental health, substance use, and sexual risk among AGYW who have left school. This study contributes to emerging knowledge on the relationship between various dimensions of abuse and risk of behavioral health problems among AGYW who are already exposed to a multitude of social determinants of mental ill-health. The findings underscore the high prevalence of physical and sexual abuse among AGYW in South Africa, with 33.5% of participants reporting exposure to physical and/or sexual abuse. These estimates are broadly consistent with those from other South African studies that report lifetime physical or sexual abuse prevalence rates ranging between 20.8% and 31.2% of AGYW [4,51,52].

Similar to findings from population and longitudinal studies from high-income countries [36,53], our findings indicate that AGYW exposed to any physical and sexual abuse are more likely to have symptoms of depression and anxiety, more problematic patterns of substance use, and greater risk of condomless sex than AGYW who have never been exposed to these forms of abuse. These findings are worrisome given the high prevalence of abuse found in this study, and because mental health and substance use concerns among AGYW go largely undetected and untreated in South Africa [3,13]. As these behavioral health problems increase AGYW’s risk for HIV [23,26], there is an urgent need to develop and implement early interventions to reduce risk of mental health and substance use problems among AGYW who have been abused. Such interventions are likely to hold both individual and societal benefits if they are integrated into comprehensive HIV prevention strategies for this key population.

Early interventions that allow for the efficient use of South Africa’s severely constrained resources for mental health and substance use services are more likely to gain traction with policymakers and service planners [32,33]. Potential strategies for the efficient use of these scarce resources include prioritizing specific types or patterns of abuse exposure associated with the greatest risk for mental health and substance use problems. Our findings shed light on whether interventions should prioritize specific types and combinations of abuse. In keeping with studies conducted with other populations [36], our findings suggest that the relationship between abuse exposure and risk for internalizing problems such as depression and anxiety seems nonspecific or unrelated to experiencing a particular type of abuse. In contrast, physical abuse (but not sexual abuse) was associated with polydrug use and substance-impaired sex. This is in keeping with earlier longitudinal studies that reported direct relationships between physical (but not sexual) abuse and substance use among adolescents [54]. As both physical and sexual abuse lead to heightened emotional reactivity and difficulties with emotion regulation, transdiagnostic mechanisms that increase risk for depression, anxiety, and substance use [55,56], interventions targeting these shared mechanisms may be effective in preventing the onset of these behavioral health problems, regardless of the kind of abuse AGYW have experienced. This is an important finding for poorly resourced settings where the capacity to implement multiple trauma-informed interventions tailored to specific type of abuse exposure is limited [57].

Our findings further suggest that AGYW exposed to multiple forms of abuse and who experience recurrent abuse may be important to prioritize for early interventions. In this study, AGYW exposed to multiple types of abuse were at greater risk for mental health and substance use concerns and sexual risk for HIV than those with a single type of abuse exposure. Risk for internalizing problems such as depression and anxiety seemed particularly elevated for AGYW exposed to multiple forms of abuse during childhood/early adolescence. Other studies from high-income settings also described the cumulative effect of exposure to multiple types of abuse on mental health outcomes [34,56]. This cumulative effect on mental health, substance use, and sexual risk is partially explained through evidence that every additional type of trauma exposure lowers the threshold for emotional reactivity, and increases emotion dysregulation [55]. Further, recurrent exposure to abuse across multiple periods of development seemed to augment risk for mental health concerns and substance-related sexual risk. In this study, a third of AGYW with histories of abuse reported abuse during childhood and recurrent abuse in late adolescence. Similar to the findings in other studies [42,43,44], these young women were at greatest risk for depression, anxiety, and substance-related sexual risk behavior.

### Limitations

Several study limitations need to be considered when interpreting these findings. First, the sample comprised AGYW recruited from 24 disadvantaged communities as part of a randomized controlled trial. Trial eligibility recruitment and geographic location may restrict the generalizability of the findings to AGYW in other parts of the country, and inferences about the prevalence of abuse in the general population of AGYW cannot be made. Second, abuse exposure was assessed retrospectively via self-report: recall and response biases may have led to these exposures being underestimated. However, questions were carefully piloted, and were asked via ACASI, a method known to improve accuracy of reporting on sensitive issues such as sexual abuse [58]. Third, although we attempted to unpack the temporality of the abuse exposures through examining the relationship between developmental timing of abuse and mental health outcomes, causality between abuse exposure and mental health, substance use, and sexual risk cannot be inferred because of the cross-sectional nature of the data. This is a particular limitation for analyses that examined the relationship between lifetime exposure and mental health symptomatology in the 90 days prior to study enrollment. Fourth, as these analyses were limited to variables included in the YWHC study, our assessment of abuse timing was crude and precluded us from examining associations between the four outcomes and abuse exposure during early childhood, a sensitive developmental period. Also, we were not able to examine associations between frequency, timing, sequencing, or chronicity of abuse, or other contextual factors, such as type of perpetrator. Including these dimensions in future longitudinal studies will allow us to examine temporal relationships, and may identify modifiable targets for interventions to reduce the impact of abuse exposure on mental health, substance use, and sexual risk outcomes for AGYW.

## 5. Conclusions

The high rates of physical and sexual abuse exposure in study participants suggest that current efforts to prevent abuse against socially vulnerable AGYW in South Africa are insufficient. Given the findings of elevated risk for mental health and substance use concerns among AGYW who have been abused, there is an urgent need to introduce and scale up evidence-based strategies for preventing all forms of interpersonal violence against children and AGYW in South Africa. This is particularly important given the dearth of treatment for the mental health sequelae of abuse in this setting. The findings of high rates of abuse recurrence and associations between abuse recurrence and greater risk to mental and behavioral health emphasize the need to develop and implement early interventions that mitigate against the short- and long-term consequences of abuse, and equip AGYW with strategies to reduce risk of recurrent exposure to violence. Additionally, consideration should be given to routinely screening AGYW for abuse exposure when they present for sexual and reproductive health services. AGYW who report histories of abuse may benefit from mental health and substance use interventions that are currently being integrated into HIV and other sexual and reproductive health services [13,15,16]. However, these interventions require adaptation to ensure they are developmentally appropriate and trauma-informed. Though all AGYW who have experienced abuse are likely to benefit from such interventions, our findings suggest that AGYW with recurrent exposure to multiple types of contact abuse are most at risk for adverse mental health, substance use, and sexual risk outcomes, and, therefore, should be prioritized for interventions. Providing trauma-informed interventions may be key to improving the psychological well-being of this population, and for reducing their risk for HIV.

## Figures and Tables

**Table 1:** Prevalence of physical and sexual abuse among young women who use substances in Cape Town, South Africa (*n* = 499).

Developmental Period	Type of Abuse Exposure			
	No Abuse Exposure (%, 95% CI) ^1^	Physical Abuse without Sexual Abuse (%, 95% CI)	Sexual Abuse without Physical Abuse (%, 95% CI)	Physical and Sexual Abuse (%, 95% CI)
Childhood or early adolescence ^2^	78.6% (74.6–82.1) *n* = 392	12.2% (9.6–15.0) *n* = 60	5.8% (4.0–8.4) *n* = 29	3.6% (2.1–6.2) *n* = 18
Late adolescence ^3^	76.2% (71.3–80.4) *n* = 380	12.2% (10.4–16.8) *n* = 66	4.0% (2.6–6.1) *n* = 20	6.6% (4.9–8.8) *n* = 33
Lifetime	66.5% (61.4–71.3) *n* = 332	18.6% (15.7–22.0) *n* = 93	6.8% (5.0–9.2) *n* = 34	8.0% (6.1–10.4) *n* = 40

^1^ 95% CI: 95% confidence intervals; ^2^ Abuse in childhood or early adolescence refers to abuse exposure before 16 years of age; ^3^ Abuse in late adolescence refers to abuse exposure in 6 months prior to study enrollment (between 16 and 19 years of age).

**Table 2:** Associations between type and combination of abuse exposure and mental health, substance use, and sexual risk among young women who use substances in Cape Town, South Africa (*n* = 499) ^1^.

Mental Health, Substance Use and Sexual Risk	Model 1: Any Abuse on CMD Risk	Model 2: Type of Abuse		Model 3: Combination of Type of Abuse		
	Any Abuse ^1^ OR (95% CI)/ *B* (95% CI) ^2,3^	Physical Abuse with/without Sexual Abuse ^1^ OR/*B* (95% CI)	Sexual Abuse with/without Physical Abuse ^1^ OR/*B* (95% CI)	Physical Abuse Only ^1^ OR/*B* (95% CI)	Sexual Abuse Only OR/*B* (95% CI)	Physical & Sexual Abuse OR/*B* (95% CI)
Depression score	1.52 ** (0.44–2.60)	1.52 * (0.31–2.80)	1.40 * (0.17–2.63)	1.03 (−0.14–2.20)	0.07 (−2.02–2.16)	3.92 *** (2.25–5.59)
Anxiety score	1.89 ** (0.67–3.11)	1.65 * (0.18–3.12)	1.59 ** (0.44–2.74)	1.43 (−0.05–2.91)	0.99 (−0.94–2.93)	3.70 *** (2.11–5.28)
Polydrug use	1.57 * (1.02–2.52)	1.68 * (1.04–2.74)	1.12 (0.59–2.13)	1.63 (0.91–2.92)	0.98 (0.40–2.40)	2.10 * (1.02–4.34)
Drug-impaired at last sex	1.64 * (1.13–2.41)	1.59 * (1.00–2.53)	1.31 (0.70–2.43)	1.60 (0.91–2.81)	1.27 (0.57–2.80)	2.17 ** (1.31–3.86)

* Significant at *p* < 0.05; ** Significant at *p* < 0.01; *** Significant at *p* < 0.001. ^1^ Series of regression equations for each outcome that adjusted for age and race; reference = no abuse exposure. ^2^ OR (odds ratio) and 95% CI (95% confidence interval) reported for logistic regression on polydrug use and impaired sex. ^3^ B, 95% CI: regression coefficient and 95% CI reported for linear regression on depression and anxiety measures.

**Table 3:** Relationship between developmental period of abuse and risk of common mental disorders among young women who use substances in Cape Town, South Africa (*n*= 499) ^1^.

Mental Health, Substance Use and Sexual Risk	Exposure during Childhood or Early Adolescence Only OR/B (95% CI) ^2,3^ (*n* = 51)	Exposure during Late Adolescence Only OR/B (95% CI) ^2,3^ (*n* = 58)	Exposure in Both Developmental Periods OR/B (95% CI) ^2,3^ (*n* = 58)
Depression score	1.14 (−0.74–3.03)	0.06 (−1.06–1.17)	3.09 *** (1.95–4.23)
Anxiety score	1.25 (−0.83–3.34)	0.76 (−0.21–1.74)	3.23 *** (1.82–4.64)
Polydrug use	0.85 (0.57–1.54)	2.13 * (1.21–3.76)	1.61 (0.87–3.00)
Drug-impaired at last sex	0.91 (0.44–1.85)	1.29 (0.64–2.59)	2.47 *** (1.51–4.07)

* Significant at *p* < 0.05; *** Significant at *p* < 0.001. ^1^ Series of regression equations for each outcome; reference = no abuse exposure. ^2^ OR (odds ratio) and 95% CI (95% confidence interval) reported for logistic regression on polydrug use and impaired sex. ^3^ B, 95% CI: regression coefficient and 95% CI reported for linear regression on depression and anxiety measures.

**Table 4:** Associations between type of abuse, developmental period of exposure, and risk of common mental disorders among young women who use substances in Cape Town, South Africa (*n* = 499) ^1^.

Mental Health, Substance Use and Sexual Risk	Physical and Sexual Abuse during Childhood or Early Adolescence ^1^ OR/*B* (95% CI) ^2,3^			Physical and Sexual Abuse in Late Adolescence ^1^ OR/*B* (95% CI)			Physical and Sexual Abuse during Both Developmental Periods OR/*B* (95% CI)		
	Physical Abuse Only	Sexual Abuse Only	Physical & Sexual Abuse	Physical Abuse Only	Sexual Abuse Only	Physical & Sexual Abuse	Physical Abuse Only	Sexual Abuse Only	Physical & Sexual Abuse
Depression score	1.81 (−0.72–3.54)	0.35 (−3.66–2.96)	4.52 ** (0.26–8.78)	−0.49 (−1.42–0.45)	−1.23 (−4.07–1.61)	0.91 (−2.41–4.23)	1.42 (−0.33–3.18)	1.16 (−2.82–5.14)	4.65 *** (3.15–6.14)
Anxiety score	1.16 (−1.79–4.11)	0.54 (−2.28–3.36)	4.45 *** (2.66–6.23)	0.04 (−1.27–1.33)	0.41 (−2.19–3.02)	0.80 (−2.58–4.19)	2.35 (−0.25–4.84)	0.54 (−4.69–5.67)	4.35 *** 2.70–6.02)
Polydrug use	1.19 (0.56–2.51)	0.59 (0.18–2.00)	0.30 (0.03–3.38)	2.70 ** (1.30–5.61)	1.14 (0.38–3.48)	2.57 (0.88–7.52)	0.92 (0.33–2.61)	0.88 (0.10–7.77)	2.37 * (1.03–5.73)
Drug-impaired at last sex	1.04 (0.49–2.21)	1.10 (0.36–3.34)	-	1.58 (0.63–3.96)	0.76 (0.15–3.73)	0.65 (0.11–3.83)	0.94 (0.37–2.33)	2.81 (0.22–11.43)	3.41 (0.73–16.23)

* Significant at *p*< 0.05; ** Significant at *p* < 0.01; *** Significant at *p* < 0.001. ^1^ Series of regression equations for each class of behavior that adjusted for age and race; reference = no abuse. ^2^ OR (odds ratio) and 95% CI (95% confidence interval) reported for logistic regression on polydrug use and impaired sex. ^3^ B, 95% CI: regression coefficient and 95% CI reported for linear regression on depression and anxiety measures.

## Data Availability

De-identified data used for this analysis are available on reasonable request from the principal investigator.

## References

[1] World Health Organization (2016). Global Health Estimates 2015: Disease Burden by Cause, Age, Sex, by Country and by Region, 2000–2015.

[2] Kuringe E., Materu J., Nyato D., Majani E., Ngeni F., Shao A., Mjungu D., Mtenga B., Nnko S., Kipingili T. (2019). Prevalence and correlates of depression and anxiety symptoms among out-of-school adolescent girls and young women in Tanzania: A cross-sectional study. PLoS ONE.

[3] Das-Munshi J., Lund C., Mathews C., Clark C., Rothon C., Stansfeld S. (2016). Mental health inequalities in adolescents growing up in post-apartheid South Africa: Cross-sectional survey, SHaW study. PLoS ONE.

[4] Myers B., Bantjes J., Lochner C., Mortier P., Kessler R.C., Stein D.J. (2021). Maltreatment during childhood and risk for common mental disorders among first year university students in South Africa. Soc. Psychiatry Psychiatr. Epidemiol..

[5] Plüddemann A., Morojele N., Myers B., Townsend L., Lombard C.J., Petersen-Williams P., Carney T., Nel E. (2014). The prevalence of risk for mental health problems among high school students in the Western Cape Province, South Africa. S. Afr. J. Psychol..

[6] Saban A., Flisher A.J., Grimsrud A., Morojele N., London. L., Williams D.R., Stein D.J. (2014). The association between substance use and common mental disorders in young adults: Results from the South African Stress and Health (SASH) survey. Pan Afr. Med. J..

[7] James S., Reddy S.P., Ellahebokus A., Sewpaul R., Naidoo P. (2017). The association between adolescent risk behaviours and feelings of sadness or hopelessness: A cross-sectional survey of South African secondary school learners. Psychol. Health Med..

[8] Kuo C., LoVette A., Stein D.J., Cluver L.D., Brown L.K., Atujuna M., Gladstone T.R., Martin J., Beardslee W. (2019). Building resilient families: Developing family interventions for preventing adolescent depression and HIV in low resource settings. Transcult. Psychiatry.

[9] Wechsberg W., Myers B., Reed E., Carney T., Emanuel A.N., Browne F.A. (2013). Substance use, gender inequity, violence and sexual risk among couples in Cape Town. Cult. Health Sex..

[10] Watt M.H., Myers B., Towe S.L., Meade C.S. (2015). The mental health experiences and needs of methamphetamine users in Cape Town: A mixed-methods study. S. Afr. Med. J..

[11] Myers B., Johnson K., Lucas W., Govender R., Manderscheid R., Williams P.P., Koch J.R. (2019). South African service users′ perceptions of patient-reported outcome and experience measures for adolescent substance use treatment: A qualitative study. Drug Alcohol Rev..

[12] Myers B., Kline L.T., Doherty A.I., Carney T., Wechsberg W.M. (2014). Perceived need for substance use treatment among young women from disadvantaged communities in Cape Town, South Africa. BMC Psychiatry.

[13] Sorsdahl K., van der Westhuizen C., Neuman M., Weiss H., Myers B. (2021). Addressing the mental health needs of adolescents in South African communities: A protocol for a randomised controlled feasibility trial. Pilot Feasibility Stud..

[14] Myers B., Carney T., Wechsberg W.M. (2016). “Not on the agenda”: A qualitative study of influences on health services use among poor young women who use drugs in Cape Town, South Africa. Int. J. Drug Policy.

[15] Myers B., Carney T., Browne F., Wechsberg W.M. (2018). Development of a trauma-informed substance use and sexual risk reduction intervention for young South African women. Patient Prefer. Adherence.

[16] Petersen Williams P., Brooke-Sumner C., Joska J., Kruger J., Vanleeuw L., Dada S., Sorsdahl K., Myers B. (2020). Young South African women on antiretroviral therapy perceptions of a psychological counselling program to reduce heavy drinking and depression. Int. J. Environ. Res. Public Health.

[17] Hobkirk A.L., Watt M.H., Myers B., Skinner D., Meade C.S. (2016). A qualitative study of methamphetamine initiation in Cape Town, South Africa. Int. J. Drug Policy.

[18] Erskine H.E., Moffitt T.E., Copeland W.E., Costello E.J., Ferrari A.J., Patton G., Degenhardt L., Vos T., Whiteford H.A., Scott J.G. (2015). A heavy burden on young minds: The global burden of mental and substance use disorders in children and youth. Psychol. Med..

[19] Fullagar S., Rich E., Francombe-Webb J., Maturo A. (2017). Digital ecologies of youth mental health: Apps, therapeutic publics and pedagogy as affective arrangements. Soc. Sci..

[20] Ngo V.K., Rubinstein A., Ganju V., Kanellis P., Loza N., Rabadan-Diehl C., Daar A.S. (2013). Grand challenges: Integrating mental health care into the non-communicable disease agenda. PLoS Med..

[21] Marinda E., Simbayi L., Zuma K., Zungu N., Moyo S., Kondlo L., Jooste S., Nadol P., Igumbor E., Dietrich C. (2020). Towards achieving the 90-90-90 HIV targets: Results from the south African 2017 national HIV survey. BMC Public Health.

[22] Abrahams N., Mhlongo S., Dunkle K., Chirwa E., Lombard C., Seedat S., Kengne A.P., Myers B., Peer N., Garcia-Moreno C. (2021). Increase in HIV incidence in women exposed to rape. AIDS.

[23] Duby Z., Appollis T.M., Jonas K., Maruping K., Dietrich J., LoVette A., Kuo C., Vanleeuw L., Mathews C. (2021). “As a young pregnant girl…The challenges you face”: Exploring the intersection between mental health and sexual and reproductive health amongst adolescent girls and young women in South Africa. AIDS Behav..

[24] Goin D.E., Pearson R.M., Craske M.G., Stein A., Pettifor A., Lippman S.A., Kahn K., Neilands T.B., Hamilton E.L., Selin A. (2020). Depression and incident HIV in adolescent girls and young women in HIV Prevention Trials Network 068: Targets for prevention and mediating factors. Am. J. Epidemiol..

[25] Mabaso M., Sokhela Z., Mohlabane N., Chibi B., Zuma K., Simbayi L. (2018). Determinants of HIV infection among adolescent girls and young women aged 15−24 years in South Africa: A 2012 population-based national household survey. BMC Public Health.

[26] Mthiyane N., Harling G., Chimbindi N., Baisley K., Seeley J., Dreyer J., Zuma T., Birdthistle I., Floyd S., McGrath N. (2021). Common mental disorders and HIV status in the context of DREAMS among adolescent girls and young women in rural KwaZulu-Natal, South Africa. BMC Public Health.

[27] Wechsberg W.M., Myers B., Kline T.L., Carney T., Browne F.A., Novak S.P. (2012). The relationship of alcohol and other drug use typologies to sex risk behaviors among vulnerable women in Cape Town, South Africa. J. AIDS Clin. Res..

[28] Wechsberg W.M., Jones H.E., Zule W.A., Myers B.J., Browne F.A., Kaufman M.R., Luseno W., Flisher A.J., Parry C.D. (2010). Methamphetamine (“tik”) use and its association with condom use among out of school females in Cape Town, South Africa. Am. J. Drug. Alcohol Abuse.

[29] Colizzi M., Lasalvia A., Ruggeri M. (2020). Prevention and early intervention in youth mental health: Is it time for a multidisciplinary and trans-diagnostic model for care?. Int. J. Ment. Health Syst..

[30] Galagali P.M., Brooks M.J. (2020). Psychological care in low-resource settings for adolescents. Clin. Child Psychol. Psychiatry.

[31] Carney T., Myers B. (2012). Effectiveness of early interventions for substance-using adolescents: Findings from a systematic review and meta-analysis. Subst. Abuse Treat. Prev. Policy.

[32] Brooke-Sumner C., Petersen-Williams P., Kruger J., Mahomed H., Myers B. (2019). ‘Doing more with less’: A qualitative investigation of perceptions of South African health service managers on implementation of health innovations. Health Policy Plan..

[33] Sorsdahl K., Naledi T., Lund C., Levitt N.S., Joska J.A., Stein D.J., Myers B. (2021). Integration of mental health counselling into chronic disease services at the primary health care level: Formative research on dedicated versus designated strategies in the Western Cape, South Africa. J. Health Serv. Res. Policy.

[34] Lindert J., von Ehrenstein O.S., Grashow R., Gal G., Braehler E., Weisskopf M.G. (2014). Sexual and physical abuse in childhood is associated with depression and anxiety over the life course: Systematic review and meta-analysis. Int. J. Public Health.

[35] Lynch S.J., Sunderland M., Newton N.C., Chapman C. (2020). Transdiagnostic risk and protective factors for psychopathology in young people: Systematic review protocol. JMIR Res. Protoc..

[36] Hughes K., Bellis M.A., Hardcastle K.A., Sethi D., Butchart A., Mikton C., Jones L., Dunne M.P. (2017). The effect of multiple adverse childhood experiences on health: A systematic review and meta-analysis. Lancet Public Health.

[37] Bonner C.P., Browne F.A., Ndirangu J.W., Howard B., Zule W.A., Speizer I.S., Kline T., Wechsberg W.M. (2019). Exploring the associations between physical and sexual gender-based violence and HIV among women who use substances in South Africa: The role of agency and alcohol. AIDS Care.

[38] Kidman R., Kohler H.P. (2019). Adverse childhood experiences, sexual debut and HIV testing among adolescents in a low-income high HIV-prevalence context. AIDS.

[39] Richter L., Komárek A., Desmond C., Celentano D., Morin S., Sweat M., Chariyalertsak S., Chingono A., Gray G., Mbwambo J. (2014). Reported physical and sexual abuse in childhood and adult HIV risk behaviour in three African countries: Findings from Project Accept (HPTN-043). AIDS Behav..

[40] Myers B., McLaughlin K.A., Wang S., Blanco C., Stein D.J. (2014). Associations between childhood adversity, adult stressful life events, and past year drug use disorders in the national epidemiological study of alcohol and related conditions (NESARC). Psycho. Addict. Behav..

[41] Schalinski I., Teicher M.H., Nischk D., Hinderer E., Müller O., Rockstroh B. (2016). Type and timing of adverse childhood experiences differentially affect severity of PTSD, dissociative and depressive symptoms in adult inpatients. BMC Psychiatry.

[42] Adams Z.W., Moreland A., Cohen J.R., Lee R.C., Hanson R.F., Danielson C.K., Self-Brown S., Briggs E.C. (2016). Polyvictimization: Latent profiles and mental health outcomes in a clinical sample of adolescents. Psychol. Violence.

[43] Dunn E.C., Soare T.W., Raffeld M.R., Busso D.S., Crawford K.M., Davis K.A., Fisher V.A., Slopen N., Smith A.D., Tiemeier H. (2018). What life course theoretical models best explain the relationship between exposure to childhood adversity and psychopathology symptoms: Recency, accumulation, or sensitive periods?. Psychol. Med..

[44] Warmingham J.M., Handley E.D., Rogosch F.A., Manly J.T., Cicchetti D. (2019). Identifying maltreatment subgroups with patterns of maltreatment subtype and chronicity: A latent class analysis approach. Child Abuse Negl..

[45] Ziobrowski H.N., Buka S.L., Austin S.B., Sullivan A.J., Horton N.J., Simone M., Field A.E. (2020). Using latent class analysis to empirically classify maltreatment according to the developmental timing, duration, and co-occurrence of abuse types. Child Abuse Negl..

[46] Wechsberg W.M., Browne F.A., Carney T., Myers B., Minnis A., MacDonald R., Ndirangu J.W., Turner L.B., Howard B.N., Rodman N. (2018). The young women′s health CoOp in Cape Town, South Africa: Study protocol for a cluster-randomised trial for adolescent women at risk for HIV. BMC Public Health.

[47] Knight K., Holcom M., Simpson D. (1998). TCU Psychosocial Functioning and Motivation Scales: Manual on Psychometric Properties.

[48] Wechsberg W.M. (1998). Revised Risk Behavior Assessment, Part I and Part II.

[49] Connor J.P., Gullo M.J., White A., Kelly A.B. (2014). Polysubstance use: Diagnostic challenges, patterns of use and health. Curr. Opin. Psychiatry.

[50] Wechsberg W.M., Jewkes R., Novak S.P., Kline T., Myers B., Browne F.A., Carney T., Lopez A.A.M., Parry C. (2013). A brief intervention for drug use, sexual risk behaviours and violence prevention with vulnerable women in South Africa: A randomised trial of the Women′s Health CoOp. BMJ Open.

[51] Optimus Study South Africa: Technical Report. Sexual Victimization of Children in South Africa: Final Report of the Optimus Foundation Study. http://www.cjcp.org.za/uploads/2/7/8/4/27845461/08_cjcp_report_2016_d.pdf.

[52] Meinck F., Cluver L.D., Boyes M.E., Loening-Voysey H. (2016). Physical, emotional and sexual adolescent abuse victimisation in South Africa: Prevalence, incidence, perpetrators and locations. J. Epidemiol. Community Health.

[53] Lewis S.J., Arseneault L., Caspi A., Fisher H.L., Matthews T., Moffitt T.E., Odgers C.L., Stahl D., Teng J.Y., Danese A. (2019). The epidemiology of trauma and post-traumatic stress disorder in a representative cohort of young people in England and Wales. Lancet Psychiatry.

[54] Benedini K.M., Fagan A.A. (2020). From child maltreatment to adolescent substance use: Different pathways for males and females?. Fem. Criminol..

[55] McLaughlin K.A., DeCross S.N., Jovanovic T., Tottenham N. (2019). Mechanisms linking childhood adversity with psychopathology: Learning as an intervention target. Behav. Res. Ther..

[56] Weissman D.G., Bitran D., Miller A.B., Schaefer J.D., Sheridan M.A., McLaughlin K.A. (2019). Difficulties with emotion regulation as a transdiagnostic mechanism linking child maltreatment with the emergence of psychopathology. Dev. Psychopathol..

[57] Myers B., Carney T., Johnson K., Browne F.A., Wechsberg W.M. (2020). Service providers′ perceptions of barriers to the implementation of trauma-focused substance use services for women in Cape Town, South Africa. Int. J. Drug Policy.

[58] Falb K., Tanner S., Asghar K., Souidi S., Mierzwa S., Assazenew A., Bakomere T., Mallinga P., Robinette K., Tibebu W. (2016). Implementation of audio-computer assisted self-interview (ACASI) among adolescent girls in humanitarian settings: Feasibility, acceptability, and lessons learned. Confl. Health.

